# A Mixed Methods Comparison of Oral Hygiene Behaviors by Gender Among Mexican-Origin Young Adults in California

**DOI:** 10.3390/oral5010005

**Published:** 2025-01-20

**Authors:** Melissa Yu, Guadalupe X. Ayala, Melody K. Schiaffino, Kristin S. Hoeft, Vanessa Malcarne, Tracy L. Finlayson

**Affiliations:** 1 NYU Langone Dental Medicine - Hawaii Island Community Health Center Advanced Education in General Dentistry, Hilo HI 96720, USA; 2 Institute for Behavioral and Community Health (IBACH), San Diego, CA 92123, USA; 3 School of Public Health, San Diego State University (SDSU), San Diego, CA 92182, USA; 4 School of Medicine, University of California San Diego (UCSD), La Jolla, CA 92093, USA; 5 School of Dentistry, University of California San Francisco (UCSF), San Francisco, CA 94143, USA; 6 Department of Psychology, San Diego State University (SDSU), San Diego, CA 92182, USA; 7 SDSU-UCSD Joint Doctoral Program in Clinical Psychology, San Diego, CA 92182, USA

**Keywords:** oral hygiene, toothbrushing, flossing, young adults, Mexican–Americans, mixed methods, qualitative

## Abstract

**Objective:**

This mixed methods study explores gender differences in, and reasons for, toothbrushing and flossing among Mexican-origin adults.

**Methods:**

Interviews and surveys about oral hygiene behaviors were collected from 72 adults (ages 21–40) living on the California–Mexico border. Interviews were audio-recorded, transcribed in their original language (English/Spanish), then coded. Survey responses were linked to coded transcripts in Dedoose. Qualitative reports were thematically analyzed for each behavior, stratified into four groups by gender and whether or not participants met American Dental Association (ADA) weekly guidelines (brushing ≥ 14/week; flossing ≥ 7/week). Self-reported weekly frequencies of brushing and flossing were collected continuously, and then dichotomized as meeting guidelines or not. Kruskal–Wallis and chi-square tests assessed differences in hygiene behavior frequency by gender. Negative binomial and logistic regressions were performed, accounting for socio-demographic characteristics.

**Results:**

Overall, 76% and 40% of adults met ADA guidelines for brushing and flossing, respectively. There were no differences in meeting ADA guidelines by gender. When brushing was examined continuously, women brushed 1.24 (1.05–1.47; *p* = 0.0099) times the rate of weekly brushing than men in the full model; flossing frequency differences were not found. Men and women, whether ADA guidelines were met or not, identified similar brushing and flossing facilitators (health concerns, aesthetics), and barriers (lack of time, not being home). Some women (mothers) were motivated to brush to be role models for their children. Self-efficacy, or confidence in ability to brush or floss, was described differently by adults who met ADA guidelines (high self-efficacy) compared to the adults not meeting guidelines (low self-efficacy).

**Conclusions:**

Integrating quantitative and qualitative data obtained from Mexican-origin adults identified few differences in both oral hygiene behaviors and the factors that influence their enactment.

## 1. Introduction

Oral diseases have become a growing concern in the United States (U.S.) over the past decades for all Americans, while disproportionately affecting racial and ethnic minorities [1,2]. About one-third (32%) of adults have untreated dental caries, and nearly half (47%) have periodontitis [3,4]. Mexican-origin adults have the highest prevalence (63%) of periodontitis compared to non-Hispanic White adults (43%) and non-Hispanic Black adults (59%) [5]. Similarly, Mexican-origin adults have the highest number of untreated dental caries among Latinos in the U.S. [6]. Untreated oral diseases can lead to pain, tooth loss, impaired ability to chew, and negatively affect quality of life [2]. The high rate of oral disease in the Mexican-origin population in the U.S. highlights a need to identify factors that influence oral hygiene behaviors and inform future intervention and prevention efforts.

Oral hygiene behaviors, specifically toothbrushing and flossing, have been linked to the reduction of common oral diseases [7]. Dental caries can be significantly reduced with effective toothbrushing [8,9]. However, brushing by itself is not enough to minimize the risk for periodontal disease [8]; using an interdental cleaning device to reduce plaque levels should be done in addition to brushing [10]. Interdental cleaning devices include dental floss, interdental brushes, wooden interdental aids, and oral irrigators [11]. The standard American Dental Association (ADA) recommendation is to brush for two minutes at least twice a day and floss once per day in order to maintain healthy gums [12]. Meeting guidelines significantly improves overall oral health by reducing common oral diseases prevalence [13].

There is a need to understand factors that influence oral health behaviors, and gender differences may be important to consider. Studies have found differences in both oral disease prevalence [14–16] and engagement in oral hygiene behaviors [17–20] by gender. Men are at higher risk of periodontal disease than women, with a 9% higher prevalence of periodontal disease compared to women [14]. Dental caries prevalence is higher among women than men [14–16]. Women more frequently brush their teeth than men [20], tend to report higher rates of brushing at least twice a day in comparison to men [21], and tend to have better oral hygiene scores [22]. Daily flossing behavior has been fairly stable in the U.S. between 2009 and 2020 in the National Health and Nutrition Examination Survey (NHANES) data, with about one in three adults reporting flossing [19,23]; men flossed less often than women according to the 2017–2018 NHANES data [18].

Oral hygiene behaviors among Mexican–American adolescents and adults have been described, but there are still gaps in understanding factors that influence engagement in these behaviors. One study of mostly Mexican–American adults in North Carolina reported that many brushed their teeth at least once a day, but had low rates of using floss; about half reported they received formal brushing and flossing instruction by a dental professional [24]. In a qualitative study of Mexican–American adolescents and adults living in the midwestern region of the U.S., nearly all reported brushing daily, but few flossed; most reported they did not receive any formal oral hygiene instruction or demonstrations from dental professionals [25]. Family upbringing and cultural influences, such as level of connection to family and length of time in the U.S., may also play a role in motivating engagement in oral hygiene behaviors [25]. Hygiene barriers that have previously been identified included lack of knowledge about how to properly floss and lack of positive influences in the environment encouraging regular implementation of hygiene behaviors [26].

The objectives of this mixed methods study were to: (1) determine if there are differences by gender in the frequency of toothbrushing and flossing among Mexican-origin adults; (2) determine if there are differences by gender in whether or not ADA guidelines for toothbrushing and flossing were met; and (3) qualitatively identify facilitators and barriers to oral hygiene behaviors, by comparing narratives across groups of young adults, stratified by gender and whether or not ADA guidelines for toothbrushing and flossing were met. Based on the patterns in the literature, it is hypothesized that differences in oral hygiene frequency will be observed in the quantitative models, with women reporting brushing and flossing more often than men. It is also hypothesized that women will be more likely to meet the ADA oral hygiene guidelines than men. Qualitative and mixed methods research often support hypothesis-generation [27–29]. A unique contribution of mixed methods research is the opportunity to connect and contextualize results of both the frequency (how often) and reasons (why) behaviors are occurring, and to generate rich understanding of complex phenomena [29].

## 2. Materials and Methods

### 2.1. Study Design

This cross-sectional study employed a mixed methods approach [28,30]. Qualitative individual interview transcripts were linked with quantitative survey data, both collected as part of the first phase of “Project Boca Sana,” a larger study of oral health behaviors and social support [31]. Interviews queried experiences and current practices with oral hygiene behaviors and dental care-seeking behaviors. There was a specific focus on querying all types of social support (e.g., instrumental, emotional) and different sources of social support (e.g., family, friends, dental care providers) for engaging in these hygiene behaviors. This study phase of Project Boca Sana focused on the open-ended, qualitative data collection efforts, and the sample was intentionally balanced by gender, marital status, and language preference (more details below). Sociodemographic characteristics and self-reported engagement in oral hygiene behaviors were collected as part of brief surveys after the interview. De-identified interview and survey data were linked by a unique participant identification number.

### 2.2. Sample

Potential participants were recruited widely in two California (CA) counties at community outreach events and through in-reach efforts among patients of the study’s two Federally Qualified Health Center (FQHC) partners at two of their clinic service sites in northern San Diego County and Imperial County, on the California–Mexico border. These regions were chosen given high proportions of Mexican-origin adults living in these California counties [32], and observed health disparities [33] and barriers to accessing dental care [34–36]. Approval for this study was granted by the San Diego State University Institutional Review Board (protocol #HS-2017-0351).

Recruitment took place in the summer and fall of 2018 by trained bilingual (English/Spanish) research assistants and clinic staff via study flyers (hard copies shared, and posted on social media), recruitment letters to community partners, word-of-mouth, and text messages and verbal announcements sent through the clinics. The opportunity to participate was advertised widely in public spaces in San Diego and Imperial Counties, California. Participant eligibility criteria included: self-identifying as a person of Mexican-origin, age between 21–40 years old, and residing in one of the two FQHC partner service areas (in northern San Diego County or Imperial County). Participants were excluded if they were pregnant or had a health condition that required pre-medication prior to their dental exams or made them unable to provide informed consent independently (see supplemental material Table S1: eligibility screening forms in English and Spanish). Adults verbally consented in their preferred language (English or Spanish) to eligibility screenings, had an opportunity to ask questions, and those interested provided written informed consent prior to participating.

Balanced sampling across several dimensions (gender, marital status, and language preference) was an important consideration in our targeted recruitment efforts. During screening and recruitment, potential participants’ characteristics were entered into a database to monitor enrollment. The goal was to enroll a sample balanced according to gender (man/woman), marital status (married/living as married, or not), and language preference (English or Spanish) in order to stratify based on these characteristics. The sample size determination considered the study’s balanced sample goals and was set at a minimum of 50 total participants (25 men and 25 women), or up to a maximum of 80 (40 men and 40 women), with about half preferring English and half preferring Spanish. This range was based on the study team’s extensive qualitative research experience, expectations for the number of interviews needed to achieve data saturation, and guidance on mixed methods in health research [30,37].

### 2.3. Interviews

A semi-structured interview guide was developed in English, translated into Spanish independently by three professional translators, then reviewed, adjudicated, and pre-tested to ensure equivalence. The interviews were conducted in the participant’s preferred language, either English or Spanish, in-person in a quiet location that was convenient for the participant (e.g., research office, FQHC office space, or reserved public library room). Trained bilingual research assistants explored topics such as perceived and received social support for oral health, engagement in oral hygiene behaviors (brushing with fluoridated toothpaste, flossing, rinsing), and dental care utilization. Probes were incorporated into the interview guide to obtain detailed responses about how and why they performed their oral hygiene habits (see supplemental material Table S2 for the semi-structured interview guide for the questions and probes in English and Spanish). Interviews each lasted about one hour.

### 2.4. Survey Measures

Brief surveys were interviewer-administered after the interviews, then participants received USD 25 cash as compensation after completing both the interview and survey. Survey questions included self-reported oral hygiene behaviors and several socio-demographic characteristics.

The outcomes of interest were self-reported frequency of oral hygiene behaviors. Participants were asked the two questions: “How many times did you brush in the past seven days?” and “Aside from brushing your teeth with a toothbrush, in the last seven days, how many times did you use dental floss or another device to clean between your teeth?”. Responses were recorded as a whole number. The frequencies were then recoded to also categorize individuals by whether or not they met ADA guidelines for brushing (≥14 times per week versus < 14) and for flossing (≥7 times per week versus < 7) based on self-report.

All sociodemographic characteristics were dichotomized. The balance characteristics were queried at the recruitment, screening, and enrollment stage. Gender (man/woman) was based on male or female sex at birth, language preference was either English or Spanish, and marital status was defined as married/living as married or single. Age was dichotomized as 21–30 versus 31–40 years old, and site was either Imperial County or San Diego County. For country of origin, participants were categorized as U.S.-born versus not U.S.-born (born in Mexico or in another country). Income was collected as <USD 30,000/annually versus ≥ USD 30,000/annually (USD 30,000 was approximately 138% U.S. federal poverty level, a meaningful income eligibility threshold for public programs). Participants’ highest level of education was dichotomized into less than high school versus completed high school or more.

### 2.5. Quantitative Analyses

Descriptive statistics were computed for each variable. To address the first research question about differences in oral hygiene behaviors by gender, two bivariate tests were conducted. A chi-square test was used to assess the relationship between gender and meeting ADA brushing or flossing guidelines. Chi-square tests also assessed if there were differences across socio-demographic characteristics by gender. The distribution of the continuous brushing and flossing counts was assessed visually using histograms, and tested for normality [38]. A Kruskal–Wallis test was selected, given the count data were not normally distributed. The Kruskal–Wallis test is the non-parametric equivalent of a one-way analysis of variance (ANOVA), and was used to assess for differences in the frequency of brushing and flossing by gender. Negative binomial regression models were conducted with the count data of brushing and flossing frequencies, adjusting for gender, marital status, preferred language, age, country of origin, income, and education. Incidence rate ratios (IRRs) and 95% confidence intervals (CI) were estimated for these models. Logistic regression models that included all the covariates assessed whether or not participants met ADA guidelines for brushing and flossing, respectively. Adjusted odds ratios (AORs) and 95% CI were estimated for the logistic regression models. There were no multicollinearity concerns in the models. Analyses were conducted using SPSS version 26.

### 2.6. Qualitative Analyses

All interviews were audio recorded, transcribed in their original language using professional transcription services, de-identified, and quality checked by a research assistant. The study team created a detailed codebook to explore the types and sources of social support for oral hygiene behaviors and dental utilization. Briefly, six bilingual coders completed training and participated in weekly meetings to ensure code comprehension and uniformity of code application. Pairs of trained coders were assigned to different sections of the codebook to apply codes to the transcripts. Dedoose^®^ version 8.0.35 qualitative software was used to code the transcripts, which allowed the codes to be co-applied to the same excerpts if there were multiple concepts present [39]. The following codes from the codebook were the focus of this qualitative analysis, to better understand the reported oral hygiene behaviors: “brushing,” “flossing,” “oral health behavior routine (current),” “facilitators/positive valence,” “barriers/negative valence,” “intrinsic motivation,” “health beliefs,” and “social norms/social pressures.”

Each transcript file was also merged with the participant’s survey data and could be filtered by gender and ADA guideline status (meeting or not meeting guidelines), creating four subgroups used for qualitative analyses. A series of reports summarizing all coded transcript excerpts were generated for each stratification group for each behavior for this thematic analysis using Dedoose. The sets of coded reports were reviewed to identify themes and better understand the facilitators and barriers to brushing and flossing across each group. This mixed methods analysis enabled in-depth exploration about the context of the reported frequency of engagement in both oral hygiene behaviors.

## 3. Results

### 3.1. Participant Characteristics

Table 1 summarizes the socio-demographic characteristics of the sample by gender which shows both men and women had similar representation by age group, marital status, and language preference. Overall, 55 (76%) met ADA brushing guidelines, and 29 (40%) met ADA flossing guidelines. There were no significant differences in meeting ADA brushing guidelines or ADA flossing guidelines by gender.

### 3.2. Brushing

**Figure 1.**
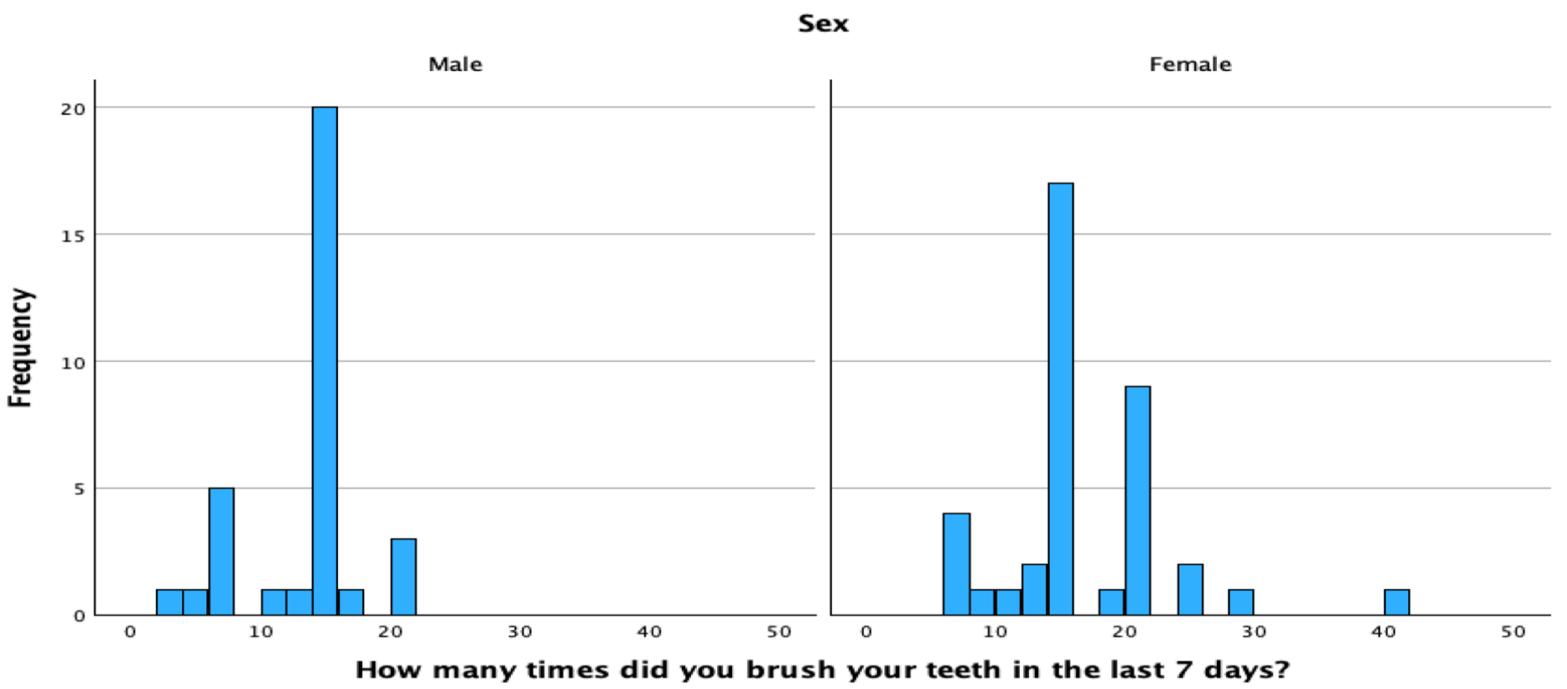
Brushing Frequency, by gender.

**Figure 2.**
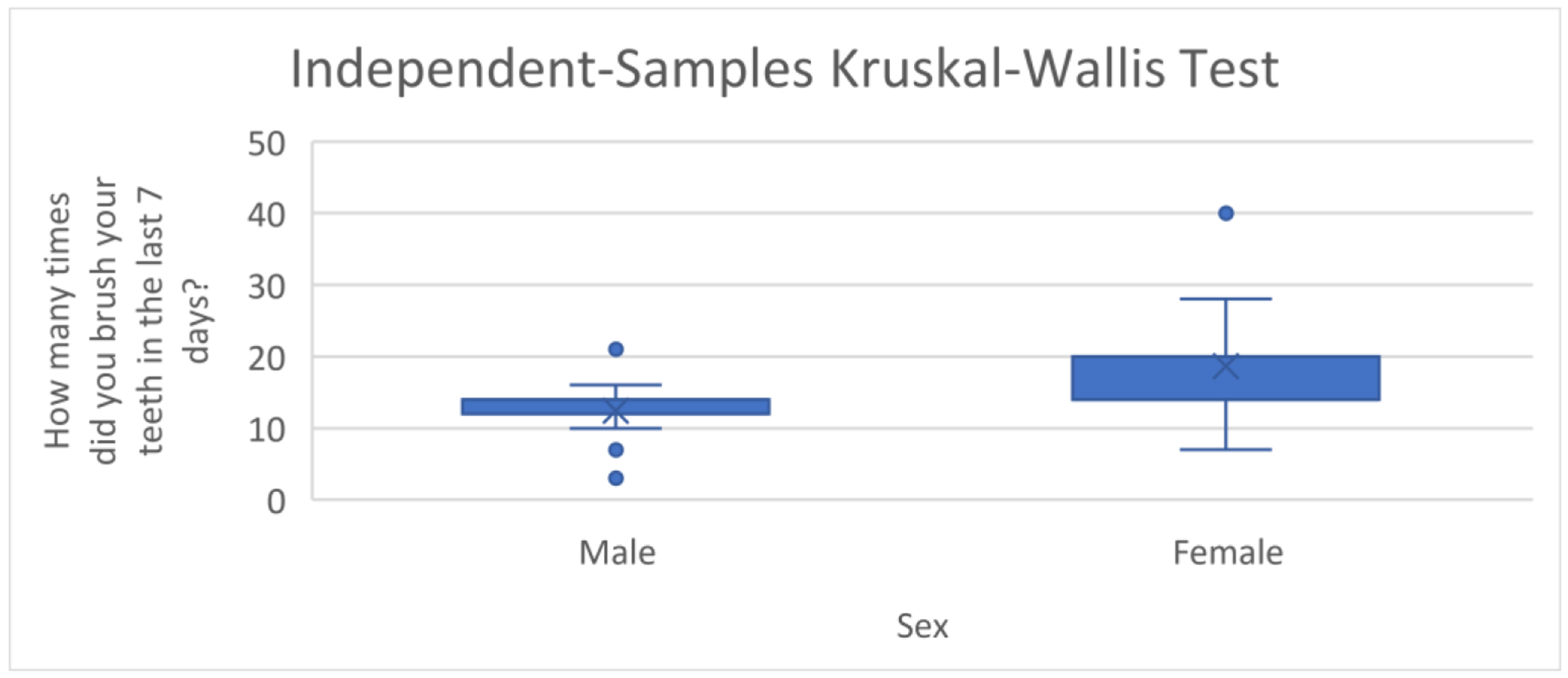
Brushing differences, by gender.

Overall, the brushing frequency ranged from 3–40 times a week, with a median of 14.00, and mean of 14.67 (standard deviation [SD] = 5.77) times in the past seven days. For men, the brushing frequency ranged from 3–21 times a week, with a median of 14.00, and mean of 12.88 (SD = 4.32) times in the past seven days. For women, the brushing frequency ranged from 7–40 times, with a median of 14.00, and mean of 16.18 (SD = 6.42) times in the past seven days; see Figure 1. While the median weekly brushing frequency was the same for both men and women, there were significant mean differences in brushing by gender (*p* = 0.040; see Figure 2). There was a wide range in the frequency distribution by gender and one participant was an outlier. However, there were still significant mean group differences by gender if the outlier was removed, so the outlier was retained. There were expected peaks at brushing about once a day (seven times/week), twice a day (14 times/week) and three times a day (21 times/week).

#### 3.2.1. Brushing Models

Table 2 presents the results of the negative binomial regression model for frequency of brushing. Gender was significantly associated with brushing frequency in the past seven days in the final adjusted model; women had 1.24 times the rate (95% CI: 1.05–1.47) of brushing than men. This model’s results support the hypothesis that women brushed more often than men.

Table 3 presents the results of the logistic regression model based on the ADA brushing guidelines. There were no significant associations in the final adjusted model, though income was marginally significant (*p* = 0.05). This model’s results do not support the hypothesis that women were more likely to meet ADA brushing guidelines than men.

In sum, only the binomial regression model for weekly brushing frequency showed significant differences in engaging in this behavior, with women brushing more often than men.

### 3.3. Flossing

**Figure 3.**
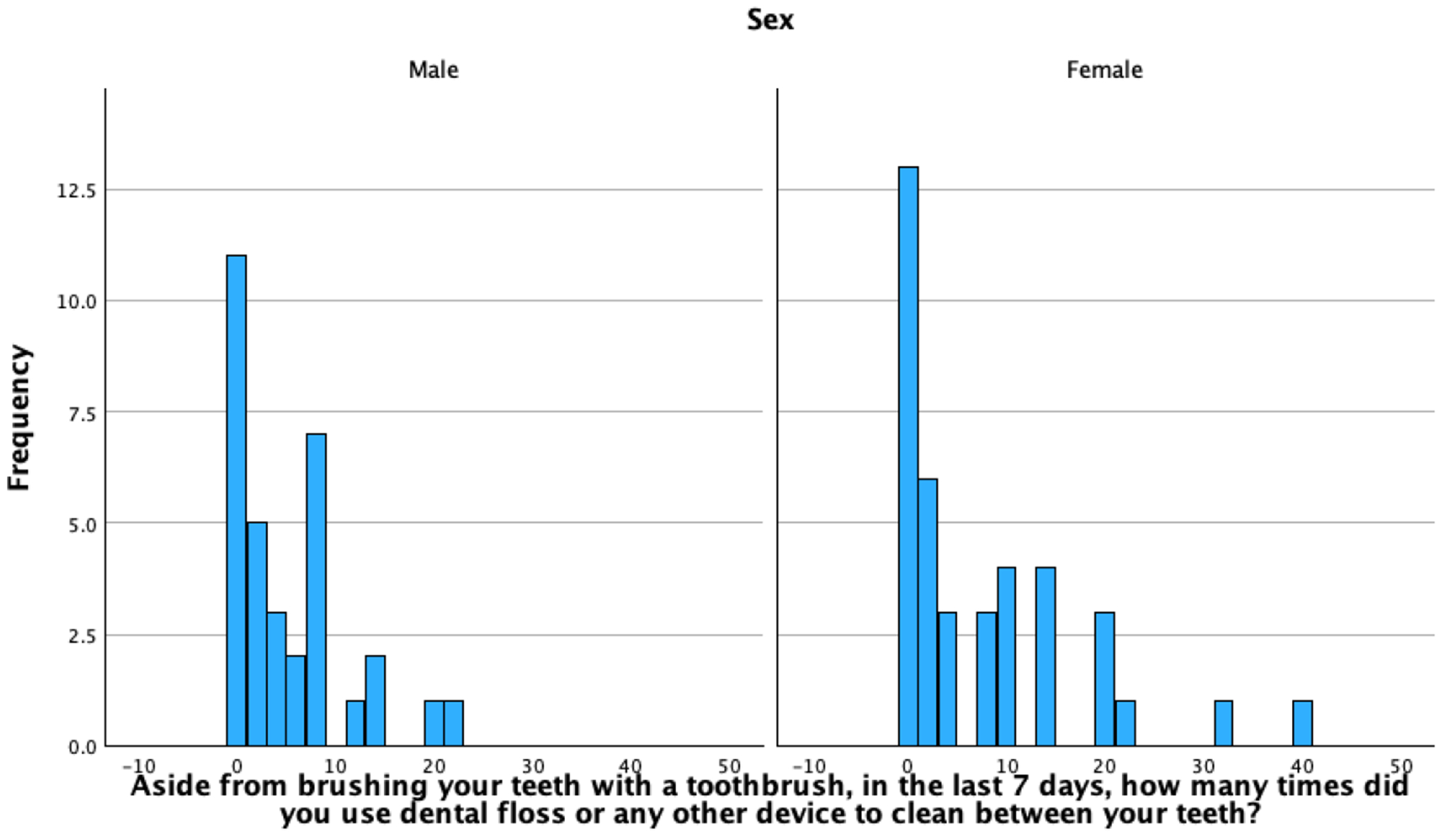
Flossing Frequency, by gender.

**Figure 4.**
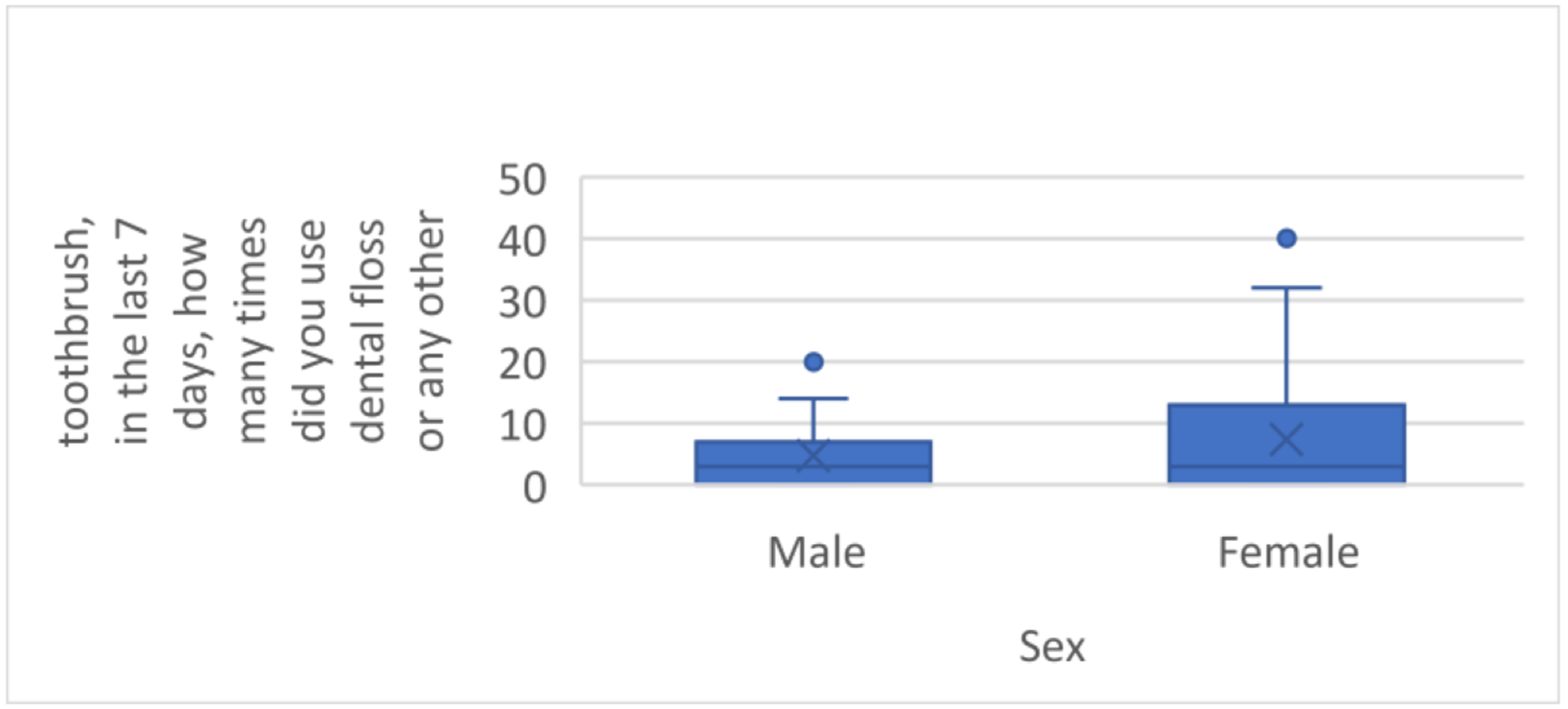
Flossing differences, by gender.

The flossing frequency ranged from 0–40 times a week, with a median of 3.00, and mean of 6.18 (SD = 8.08) times in the past seven days. For men, flossing frequency ranged 0–21 times a week, with a median of 3.00, and mean of 4.79 (SD = 5.72). For women, flossing frequency ranged 0–40 times, with a median of 3.00, and mean of 7.36 (SD = 5.72); see Figure 3. There were no significant differences in flossing by gender (*p* = 0.500); see Figure 4.

#### 3.3.1. Flossing Models

Table 4 presents the results of the negative binomial regression model for frequency of flossing. There were no significant differences in flossing frequency in the past seven days by gender. Spanish-speaking adults had a significantly higher rate of flossing in the past seven days, with 1.46 times the incidence rate (95% CI: 0.61–3.52) of flossing than English-speaking adults. This model’s results do not support the hypothesis that women flossed more often than men.

Table 5 presents the results of the logistic regression model for meeting ADA flossing guidelines. Gender was not significantly associated with meeting ADA flossing guidelines. Adults living in Imperial County were 3.89 times (95% CI: 1.16–13.08) more likely than adults living in San Diego County to meet ADA flossing guidelines. This model’s results do not support the hypothesis that women were more likely to meet ADA guidelines for flossing than men.

In sum, none of the flossing models indicated differences in frequency of engaging in this behavior between men and women. Flossing was a less common behavior overall than brushing, for both men and women. Qualitative results about oral hygiene behaviors are presented in the next section, first for brushing, then flossing.

### 3.4. Qualitative Results

Several common themes were described by all four groups (determined using the quantitative data for whether or not men and women met ADA oral hygiene guidelines). Results are presented divided by brushing and flossing, with example quotations provided that represent the common points made by participants in the group. Qualitative data were compared between the four groups in the analysis process, but are presented together when there were no differences.

#### 3.4.1. Brushing

Table 6 presents a summary of the brushing themes, which are described in further detail below with supporting illustrative quotes.

#### 3.4.2. Brushing Facilitators

Preventing disease and negative health consequences were described as primary motivations for regular brushing by all groups. This included brushing regularly for health reasons to avoid negative consequences such as cavities, plaque, root canals, dental pain and/or losing teeth. Aesthetic reasons were also mentioned by all groups, often in the same line of reasoning as disease prevention. Aesthetic motivations included desiring white teeth and good breath.

*[I brush regularly] for dental hygiene. I guess just to prevent my mouth from smelling bad, or to keep my teeth white, to prevent any disease or toothaches. I don’t want to get pains in my mouth. I don’t want to get … I don’t know. I didn’t want to have crooked teeth or anything like that, but yeah, just for health reasons, you know?* 21-year-old English-speaking man, does not meet ADA guidelines

*To avoid cavities, plaque, [and] to avoid losing teeth that kill the roots, hmm, what else? To avoid infections because you can also have mouth infections*. 40-year-old Spanish-speaking man, meets ADA guidelines

*[I brush regularly to] not have cavities, and it makes me feel better also because you have cleaner and whiter teeth*. 39-year-old English-speaking woman, does not meet ADA guidelines

Men and women who met ADA guidelines mentioned having oral hygiene routines that included regular and thorough brushing.

*Well, I start [my routine] by, I like to abide by that old rule, at least two minutes brushing your teeth because that’s something my mom always told me growing up. You have to brush your teeth at least two minutes for it to be effective. Then growing up, she [mom] would always tell me like, you should have brush your tongue and the roof of your mouth because a lot of germs accumulate there so I try to do that… Brushing, brushing… I also like to try to brush the gums really well just because the dentist recommends it whenever I see her*. 24-year-old English-speaking woman, meets ADA guidelines

*Now as I’m older I have more of a routine that [includes brushing] after I eat, ahh!, in the morning of course, or before going to sleep too*. 38-year-old Spanish-speaking man, meets ADA guidelines

Women who met ADA guidelines were the only group that said that they brushed to set a good example for their children.

*Well what helps me is setting an example for my children. That they brush their teeth and because I’ve already lost a tooth, for that reason, I teach them and tell them. Then, my tooth broke in the middle and I said “Look, I don’t want what happened to me to happen to you.” I mean, I always remember [to brush] because I want my children to have healthy teeth*. 40-year-old Spanish-speaking woman, meets ADA guidelines

#### 3.4.3. Brushing Barriers

All groups mentioned not being at home as a barrier to brushing regularly. This included long work schedules that kept them out all day, vacations, and other situations in which they were in an unfamiliar environment without their usual oral hygiene supplies.

*Sometimes when I go on trips, I stay at a hotel, sometimes I’m like, oh I forgot my toothbrush. And when I go out of the house, overnight, and I forget my toothbrush, that’s the one time that’s harder to brush my teeth*. 25-year-old English-speaking man, meets ADA guidelines

*Yeah, like when I’m on vacation with family, friends. Sometimes it’s hard to, like if you forget to pack it [toothbrush], then you have to find someplace to buy a toothbrush or let’s say you’re on the road, it’s really hard because we like to take road trips instead of flying, so if we’re on the road, it’s like, okay, how are we going to brush our teeth? We have to make sure we have a water bottle and we have to make sure that it’s readily on hand. If we’re staying at someone’s house and we forget, again if we forget to pack, you have to try to ask politely to see if they have any spare toothpaste or spare floss. Yeah, it’s harder when you’re not at home I guess*. 24-year-old English-speaking woman, meets ADA guidelines

Groups that did not meet ADA guidelines mentioned additional barriers to brushing, including lack of time and forgetting.

*It’s just sometimes when I work really early and I’m used to working late, I skip it [brushing my teeth].* 21-year-old English-speaking man, does not meet ADA guidelines

*For example, in the morning sometimes I just don’t have the time because I get up in the morning, I have to clean and I’m in a hurry because sometimes it’s already time to make lunch and I have no time to do it. Then, I’m getting the kids ready for school and there is no time. Then my priority is to do what needs to be done and not to brush my teeth sometimes, this is when I can’t [brush my teeth]*. 31-year-old Spanish-speaking woman, does not meet ADA guidelines

Some men who did not meet ADA guidelines also mentioned dental pain as a barrier to brushing.

*Yeah. On my right side, my back teeth, I have cavities, so sometimes it hurts, and it’s difficult [to brush].* 34-year-old English-speaking man, does not meet ADA guidelines

#### 3.4.4. Brushing Self-Efficacy

While participants did not use the term self-efficacy, brushing self-efficacy (the confidence in one’s ability to brush) was illustrated and described through participants’ stories and examples. Some participants thought they brushed well, while others were unsure, and a few admitted they did not think they brushed well at all. High self-efficacy was more common among men and women who met ADA guidelines.

*I feel like because I don’t brush my teeth as often as before maybe I’m doing it wrong now. But yeah, I think I do it right. I just don’t do it with as much energy, I guess, or as much willingness. I kind of see it like a chore now, yeah.* 21-year-old English-speaking man, does not meet ADA guidelines

*To be honest, I just do it [brush] until I feel that I’ve got whatever I need to get. I mean, I don’t know if I’m doing [brushing] right or wrong.* 21-year-old English-speaking woman, does not meet ADA guidelines

#### 3.4.5. Flossing

Table 7 presents a summary of flossing themes, which are described in further detail below with supporting illustrative quotes.

#### 3.4.6. Flossing Facilitators

Across all groups, using floss to remove food particles caught between teeth was mentioned as the primary purpose and motivation for flossing, whether they met ADA flossing guidelines or not. Many adults in all groups also expressed that they knew brushing alone did not clean their mouth entirely, and believed flossing helped prevent gingivitis and bleeding gums.

*The dentist told me that I needed to start flossing, that I needed to floss because I guess food goes into, I guess next to your gums and that’s what made it … Well, I guess that’s what was causing the bleeding. I wasn’t cleaning between my teeth and so I saw the importance of it because every time I would brush my teeth I would see blood and that was the cause of it.* 30-year-old English-speaking man, does not meet ADA guidelines

*It [flossing] gets all the stuff that gets in between the teeth. Like pieces of little strings of meat, popcorn mostly … I have popcorn stuck in my mouth, and sometimes [my boyfriend] goes, oh just let it [stay] there, and I’m like no, get floss or a pick or whatever you can use and get it out. And I think there’s all that stuff that’s in there can make plaque or something, it gets loose so when you brush your teeth, you pretty much get it out or help with it.* 25-year-old English-speaking man, meets ADA guidelines

*More than anything, [I believe flossing helps with] taking away my food [stuck between my teeth], and I have seen that… I do not have as much plaque.* 40-year-old Spanish-speaking woman, does not meet ADA guidelines

*It [flossing] helps with getting any bacteria buildup out, any plaque buildup, any food particles. I mentioned earlier, the coating on your tooth it’s like that soft weird coating, it helps remove all that, any build up you might have on your teeth, it helps with it for sure especially your back teeth because it’s hard to get back in there sometimes, on all the little crevices with your toothbrush so floss really helps remove anything that your toothbrush might have missed.* 24-year-old English-speaking woman, meets ADA guidelines

Men and women who met ADA guidelines mentioned having more oral hygiene routines that consistently included flossing.

*When I finish brushing my teeth, I also have at work from time to time, when I stop- when I eat my lunch, when I’m done I go and floss so I can remove any little things that are left in my teeth. I do have a package at work just in case.* 26-year-old Spanish-speaking man, meets ADA guidelines

*Well, I like to keep it consistent. So, I’m like, “Okay, I really don’t want to [floss] today because I want to go to bed. But I already did for like two weeks, like I have to keep doing it.” You know?… I think just the routine. It’s always like the hardest to start. And then, after that, if you just keep going, it gets a little easier. But I think just the routine. And I told my boyfriend and my sister. And then, I just kept going [flossing all my teeth] for a long time.* 22-year-old English-speaking woman, meets ADA guidelines

*It’s [flossing] just all part of the routine now… I don’t see it so much as a chore as I used to when I was younger because again when you’re little, you’re just like oh, do I really have to floss? Is it that important? Once you just get in the habit of it, it’s just so routine. You feel weird not doing it after a while.* 24-year-old English-speaking woman, meets ADA guidelines

Among adults meeting guidelines that flossed as part of their daily routine, some mentioned using alternatives to traditional floss such as a floss pick or oral irrigator. Some also noted that it was helpful to keep them out on their sinks where they were visible, and to carry floss picks with them so they were readily available for use.

*Yeah, mainly that’s the one I use [floss picks]. I don’t really use the string ones because I feel like I don’t know how to do it.* 21-year-old English-speaking woman, meets ADA guidelines

*If I’m going to do the front teeth I might as well get the back teeth too, so then I was looking through the dental aisle, and I was like, oh, this [dental picks] looks very helpful and then I tried it out, and I’ve stuck with it since.* 25-year-old English-speaking man, meets ADA guidelines

*I have the one [Waterpik device] that I have in the bathroom and we have the one that, when we travel or whatever, we take one [floss picks with hooks] too.* 21-year-old Spanish-speaking man, meets ADA guidelines

#### 3.4.7. Flossing Barriers

Not being at home was a barrier to flossing mentioned by all adults across all groups.

*When you’re not at home and sometimes you don’t take something with you to floss. Then that is more difficult for you.* 31-year-old Spanish-speaking woman, meets ADA guidelines

Adults who did not meet guidelines found it more difficult to floss when they were in front of other people. This group also frequently mentioned forgetting to floss, not having time to build flossing into their routines, and disliking the sensations on their gums they felt when using floss.

*Well in front of people, no, no it is not good to use a floss pick because that is something like hygiene and it is as if you went to the bathroom in front of people, right?* 40-year-old Spanish-speaking man, meets ADA guidelines

*Just to remind like, “I need to do this.” But like I said, sometimes I forget because of my hours at work. I’m in a hurry and I’m like, “Let’s go. Let’s just go.” Sometimes, like I said, I’ll forget to do flossing. I’ll just continue brushing my teeth and mouthwash and that’s it*. 35-year-old English-speaking woman, does not meet ADA guidelines

*[flossing] it’s not really something that I do … I don’t have a routine for [flossing]. I know for flossing one of the reasons why I don’t do it that often, it’s not there. It’s not visible. Usually my flosses are separate, they’re put away. So maybe that might be the reason why I don’t floss every day…* 30-year-old English-speaking man, does not meet ADA guidelines

*The sensation more than anything I don’t like, I have used it [floss], but no more than a couple times, twice at the most. Like the feeling that I feel like something is cutting me, I don’t know, I don’t know if I have very sensitive gums, but I feel, I don’t like it.* 38-year-old Spanish-speaking man, does not meet ADA guidelines

*But for me, it’s really hard to use it [floss]. I don’t know the way to use it or I haven’t had the proper way to use it or someone to show me how to use it, that’s why I don’t like to use it. Because I hurt myself once [while flossing] and I just don’t like that feeling of hurting.* 35-year-old English-speaking woman, does not meet ADA guidelines

Women who did not meet guidelines were the only group that also mentioned they did not floss due to lack of time. Men who did not meet guidelines were the only group who also mentioned they did not floss due to laziness.

#### 3.4.8. Flossing Self-Efficacy

Self-efficacy, the confidence in one’s ability to floss, was a relevant factor that varied by group. Men and women meeting flossing guidelines more often described having higher flossing self-efficacy, along with noting that flossing was a part of their regular daily routines. Adults in the group who did not meet guidelines indicated that their lack of flossing ability was a deterrent.

*No [I don’t think I floss well], I only use it on my front teeth and it is work for me to use it. So I don’t know how to use it [floss].* 27-year-old English-speaking woman, does not meet ADA guidelines

## 4. Discussion

In the quantitative analysis, gender differences were only found in one regression model, when toothbrushing was treated as a continuous variable, and women brushed significantly more often than men in the last seven days. This was the only model in which the hypothesis was supported; in the other three regression models the hypotheses were not supported. No differences in flossing frequency by gender were found. No differences by gender were found in the bivariate or multivariable models when either hygiene behavior was assessed in terms of meeting ADA guidelines. Using ADA guidelines to stratify and explore differences by gender in the qualitative analysis revealed consistent patterns in the themes identified. There were several common facilitators and barriers identified by men and women. All adults meeting ADA guidelines for brushing and flossing were more motivated to prevent disease and expressed higher self-efficacy. Not being home was a common barrier to performing oral hygiene among adults not meeting guidelines. Disliking the flossing sensation was another common barrier.

The finding that women brushed more often than men aligns with the toothbrushing literature, but the lack of differences by gender in flossing conflicts with results from other flossing studies [17–20]. Previous research provides some possible explanations for the observed differences by gender in brushing frequency. Women tend to be more accepting of help-seeking behaviors and more compliant with dental recommendations, such as the regular performance of hygiene behaviors [21]. Similarly, women tend to have greater knowledge and more positive attitudes towards their oral health and more engagement in oral hygiene [15]. Women also often take care of other family members’ health, particularly if they are mothers and primary caregivers for young children. One unique theme that emerged in the qualitative results by some women (who were mothers meeting ADA brushing guidelines) was that they were motivated to brush regularly to be role models for their children, which has been reported in other studies [40]. Another possibility is that mothers may be at home with young children more often, and being at home was identified as making it easier to engage in oral hygiene behaviors regularly.

The lack of differences by gender in flossing frequency or meeting flossing guidelines differed from previous research [15,19]. This could be due in part to the different ways flossing frequency was queried. The NHANES 2011–2014 survey question asked about number of days flossed in the last seven days, with possible response options ranging zero to seven days. Fleming and colleagues grouped adults into non-flossers (flossing zero days), those with some flossing (one to six days), or daily flossers (seven days) [19]. This study’s survey queried the number of times flossed in the last seven days, and did not restrict response options, yielding a wide range of responses. Dichotomizing responses using ADA guidelines may have inhibited our ability to detect differences between the two groups. The lack of significant differences by gender in flossing also could be related to low rates of flossing generally, though rates in this sample were similar to reported adult U.S. rates [19].

This study employed two operationalizations of oral hygiene behavior and found differences by gender with the continuous weekly frequency, but not when using the dichotomization related to meeting ADA guideline. The ADA brushing guidelines themselves have their limitations. A literature review investigated how often individuals should be brushing their teeth and it was revealed that brushing once a day, if done meticiulously, is sufficient to prevent dental caries and periodontal disease [41]. An article that the ADA cites to support its brushing guidelines highlights that it is the length of time spent brushing, and not the frequency, that increases plaque removal [13]. Future research should investigate if there are differences by gender when it comes to length of time spent brushing their teeth and effectiveness of plaque removal.

The qualitative findings of this study about brushing were similar to the one other study by Aguirre-Zero and colleagues [26] that investigated oral hygiene themes in the Mexican-origin population. Similarly, the majority of this study’s sample acknowledged the importance of regular brushing, even though not everyone brushed twice/day in the past week. There were shared identified barriers for brushing regularly, including work and school schedules causing a lack of time. Relatively few adults did not meet ADA brushing guidelines. More brushing barriers were mentioned by adults meeting ADA guidelines in this study, and men noted that current dental problems and pain were a deterrent. Dental problems and pain are indicators of a need for dental services; men delaying dental care to address problems and alleviate pain may be due to challenges in accessing care. Dental visits also provide oral hygiene instruction and demonstrations, and dental supplies (new toothbrushes and floss) to support continued regular oral hygiene at home between visits.

The qualitative findings of this study about flossing revealed there was not a consensus on the importance of flossing regularly. More than half the adults in this study did not meet ADA guidelines. Flossing was generally viewed more as an occasional activity that was needed when food particles got stuck in between teeth. This is consistent with findings by Aguirre-Zero and colleagues [26] that flossing was a need-driven behavior rather than a habitual one. A common barrier to flossing mentioned by all adults that did not meet flossing guidelines was dislike of the flossing sensation or feeling. This discomfort could be related to not being familiar with proper flossing technique or could be reduced with the use of other types of interdental cleaning devices. Several expressed not knowing correct flossing technique, and this lack of knowledge and confidence contributed to keeping flossing out of their regular hygiene routines.

Self-efficacy, or confidence in ability to perform a behavior, is a powerful and influential perception. There were some mixed comments related to self-efficacy in the qualitative analysis, but there appeared to be general patterns with regular brushers and flossers being more sure about their abilities. In previous research, women demonstrated higher levels of oral hygiene-related self-efficacy than men, which predicted their positive oral hygiene outcomes [42]. In a study of Canadian adolescents, girls had higher brushing self-efficacy, which was positively associated with more frequent brushing [43]. Teaching proper oral hygiene technique to young adults can enhance their skills and confidence to perform both brushing and flossing regularly. Educating young adults about the different options for interdental cleaning devices can also encourage the behavior and promote oral health. A study using NHANES 2011–2014 data found that more frequent use of interdental cleaners was correlated with better clinical oral health indicators for periodontal disease and caries [44].

### 4.1. Strengths and Limitations

This study has several strengths and limitations that must be considered when interpreting results. A study strength was the integrated mixed methods approach. Linking survey responses with rich narratives enabled an opportunity to explore and contextualize findings. Using the closed-ended responses to stratify and create meaningful groups to answer the research questions was also a strength of this study. Survey data alone do not provide “how” or “why” information to help interpret results. Identified themes in the qualitative analysis highlighted similarities and some differences by gender in facilitators and barriers to engaging in oral hygiene behaviors. The sample size was large for a qualitative analysis and this study makes a contribution by focusing on young adults between ages 21–40. In addition, the sample was unique in that it was balanced by gender which strengthened the findings of oral hygiene differences and supported stratifying by more than one characteristic to make meaningful comparisons by gender and meeting ADA guidelines.

Limitations included that the sample size was likely underpowered and skewed for some categories and the quantitative analysis in particular would have benefited from a larger sample. This convenience sample was drawn from the California–Mexico border region, thus results may not be generalizable to other Mexican–American populations in other regions of the U.S. or elsewhere. Another limitation was the survey data relied on self-report of oral hygiene behavior in the last seven days, which is prone to both social desirability and recall biases. Self-reported data on the frequency of engaging in these behaviors do not provide information about the quality of brushing and flossing, or if it was effective in removing plaque. No clinical assessment data were collected in this study. A further limitation of the survey was the use of single-item measures making it challenging to assess their construct validity.

### 4.2. Conclusions

Most adults in this sample brushed regularly (76% met ADA guidelines of twice/day), but not as many reported flossing regularly (only 40% met ADA guidelines of once/day). There were no differences by gender in whether or not ADA oral hygiene guidelines were met. Differences by gender were observed for continuous toothbrushing frequency, and Mexican-origin young adult women brushed more frequently than men in the last seven days. No differences by gender were observed for flossing frequency in the last seven days. Qualitative results provide insights about patterns of facilitators and barriers to engaging in regular oral hygiene behaviors, and have implications for promoting regular brushing and flossing among Mexican-origin young adults. This study has highlighted areas that merit further investigation. There is potential for future interventions to promote flossing and interdental cleaning efforts to reduce oral disease prevalence in the Mexican-origin population.

## Supplementary Material

Supplement 1.screening form

Supplement 2.interview guide

## Figures and Tables

**Table 1.** Participant Characteristics (n= 72).

	Men	Women	Total	
	n = 33	n = 39	n = 72	
	n (%)	n (%)	n (%)	*p*-Value
**Age**				
21–30 years	15 (45%)	17 (44%)	32 (44%)	
31–40 years	18 (55%)	22 (56%)	40 (56%)	0.050
Marital Status				
Not married	18 (55%)	19 (49%)	37 (51%)	
Married	15 (45%)	20 (51%)	35 (49%)	0.243
Language				
English	16 (48%)	17 (44%)	33 (46%)	
Spanish	17 (52%)	22 (56%)	39 (54%)	0.173
Country of origin				
U.S.-born	18 (55%)	14 (36%)	32 (44%)	
Not U.S.-born	15 (45%)	25 (64%)	40 (56%)	0.113
Site				
Imperial County	18 (55%)	22 (56%)	40 (56%)	
San Diego County	15 (45%)	17 (44%)	32 (44%)	0.874
Income ^1^				
<USD 30,000/year	16 (48%)	18 (55%)	40 (56%)	
≥USD 30,000/year	15 (51%)	15 (38%)	30 (43%)	0.405
Education				
Less than high school	4 (12%)	7 (18%)	11 (15%)	
High school or more	29 (88%)	32 (82%)	61 (85%)	0.492
ADA Brushing Guidelines ^2^				
Met (≥14 times/week)	24 (73%)	31 (80%)	55 (76%)	
Not Met	9 (27%)	8 (20%)	17 (24%)	0.501
ADA Flossing Guidelines ^3^				
Met (≥7 times/week)	12 (36%)	17 (44%)	29 (40%)	
Not Met	21 (64%)	22 (56%)	43 (60%)	0.533

1 Two men responded “Don’t Know” and were excluded from the chi-square test.

2 Participants in each of the four groups for the qualitative analysis for brushing, based on American Dental Association (ADA) guidelines.

3 Participants in each of the four groups for the qualitative analysis for flossing, based on American Dental Association (ADA) guidelines.

**Table 2.** Negative Binomial Regression Model for Brushing Frequency (n = 72).

	IRR ^2^ (95% CI)	*p*-Value
Age: 31–40 years	1.04 (0.86–1.26)	0.6992
Gender: Women	1.24 (1.05–1.47)	0.0099
Marital: Married	1.00 (0.83–1.21)	0.9913
Lang: Spanish	1.18 (0.97–1.44)	0.0900
Origin: U.S.-born	0.95 (0.79–1.15)	0.6041
Site: Imperial County	0.90 (0.75–1.09)	0.2878
Income ^1^: <USD 30,000/year	1.12 (0.93–1.33)	0.2560
Educ: <high school	1.19 (0.70–2.03)	0.5103

Reference groups: 21–30 years, men, not married, English, not U.S.-born, San Diego County, ≥30,000, ≥HS.

1 Two men that responded “Don’t Know” were in the reference category to maintain sample size.

2 Frequency of brushing in the last seven days. IRR = Incidence Rate Ratio. CI = confidence interval.

**Table 3.** Logistic Regression Model for Meeting ADA Brushing Guidelines (n = 72).

	AOR ^2^ (95% CI)	*p*-Value
Age: 31–40 years	0.59 (1.40–2.49)	0.4732
Gender: Women	0.74 (0.22–2.53)	0.6277
Marital: Married	3.05 (0.70–13.29)	0.1374
Lang: Spanish	0.42 (0.09–1.93)	0.2652
Origin: U.S.-born	1.01 (0.24–4.16)	0.9934
Site: Imperial County	2.05 (0.47–8.88)	0.3395
Income ^1^: <USD 30,000/year	0.26 (0.07–1.01)	0.0512
**Educ**: <high school	0.45 (0.05–3.94)	0.4697

Reference groups: 21–30 years, men, not married, English, not U.S.-born, San Diego County, ≥30,000, ≥HS.

1 Two men that responded “Don’t Know” were grouped with reference category to maintain sample size.

2 Meeting American Dental Association (ADA) Brushing Guidelines = brushing ≥ 14 times in the last seven days. AOR = Adjusted Odds Ratio, CI = Confidence Interval.

**Table 4.** Negative Binomial Regression Model for Flossing Frequency (n = 72).

	IRR ^2^ (95% CI)	*p*-Value
Age: 31–40 year	0.93 (0.38–2.26)	0.8654
Gender: Women	1.53 (0.76–3.05)	0.2299
Marital: Married	1.46 (0.61–3.52)	0.3953
Lang: Spanish	2.47 (1.07–5.68)	0.0335
Origin: U.S.-born	0.53 (0.23–1.26)	0.1498
Site: Imperial County	0.55 (0.24–1.27)	0.1638
Income ^1^: <USD 30,000/year	1.70 (0.84–3.45)	0.1388
Educ: <high school	2.77 (0.27–28.23)	0.3899

Reference groups: 21–30 years, men, not married, English, not U.S.-born, San Diego County, ≥30,000, ≥HS.

1 Two men that responded “Don’t Know” were in the reference category to maintain sample size.

2 Frequency of brushing in the last seven days. IRR = Incidence Rate Ratio. CI = confidence interval.

**Table 5.** Logistic Regression Model for Meeting ADA Flossing Guidelines (n = 72).

	AOR ^2^ (95% CI)	*p*-value
Age: 31–40 years	1.27 (0.39–4.17)	0.0692
Gender: Women	0.75 (0.25–2.20)	0.5938
Marital: Married	0.39 (0.12–1.28)	0.1211
Lang: Spanish	0.36 (0.10–1.35)	0.1304
Origin: U.S.-born	0.94 (0.27–3.23)	0.9214
Site: Imperial County	3.89 (1.16–13.08)	0.0282
Income ^1^: <USD 30,000/year	0.64 (0.21–1.98)	0.4388
Educ: <high school	0.27 (0.05–1.50)	0.1346

Reference groups: 21–30 years, men, not married, English, not U.S.-born, San Diego County, ≥30,000, ≥HS.

1 Two men that responded “Don’t Know” were grouped with reference category to maintain sample size.

2 Meeting American Dental Association (ADA) Brushing Guidelines = brushing ≥ 14 times in the last seven days. AOR = Adjusted Odds Ratio, CI = Confidence Interval.

**Table 6.** Summary of Brushing Themes.

	Men		Females	
Brushing ^1^	Does not meet ADA guidelines (n = 9)	Meets ADA guidelines (n = 24)	Does not meet ADA guidelines (n = 8)	Meets ADA guidelines (n = 31)
Facilitators	- Motivated to prevent future dental disease - Motivated to prevent bad breath	- Motivated to prevent future dental disease - Motivated for aesthetic reasons	- Motivated to prevent future dental disease - Motivated for aesthetic reasons	- Motivated to prevent future dental disease - Motivated to be a role model for children
Barriers	- Not at home - Current dental problems/pain - Lack time	- Not at home	- Forget - Lack time	- Not at home
Self-efficacy	- Low self-efficacy	- High self-efficacy, and part of routine	- Low self-efficacy	- High self-efficacy, and part of routine

1 Meeting American Dental Association (ADA) guidelines for brushing ≥14 times/week.

**Table 7.** Summary of Flossing Themes.

	Men		Women	
Flossing ^1^	Do not meet guidelines (n = 21)	Meet guidelines (n = 12)	Do not meet guidelines (n = 22)	Meet guidelines (n = 17)
Facilitators	- Motivated to clean between teeth, and remove food particles	- Motivated to clean between teeth, and remove food particles	- Motivated to clean between teeth, and remove food particles	- Motivated to clean between teeth, and remove food particles
Barriers	- Forget - Dislike the sensation	- Not at home - Not in front of others	- Lack time or forget - Dislike the sensation	- Not at home
Self-efficacy	- Low self-efficacy, no routine	- High self-efficacy, part of routine	- Low self-efficacy	- High self-efficacy, part of routine

1 Meeting American Dental Association (ADA) guidelines for flossing ≥ 7 times/week.

## Data Availability

The datasets presented in this article are not publicly available because participants did not consent to data being made publicly available. Reasonable requests to access limited de-identified datasets should be directed to the study PI, Dr. Tracy Finlayson at tfinlays@sdsu.edu.
